# Measurement of Ether Phospholipids in Human Plasma with HPLC–ELSD and LC/ESI–MS After Hydrolysis of Plasma with Phospholipase A_1_

**DOI:** 10.1007/s11745-016-4170-9

**Published:** 2016-07-07

**Authors:** Shiro Mawatari, Seira Hazeyama, Takehiko Fujino

**Affiliations:** Institute of Rheological Functions of Food, 2241 Kubara, Hisayama Chou, Kasuya-gun, Fukuoka, 811-2501 Japan

**Keywords:** Ether phospholipids, Human plasma, High-performance liquid chromatography, Evaporative light scattering detector, Electrospray ionization detector

## Abstract

Ethanolamine ether phospholipid (eEtnGpl) and choline ether phospholipid (eChoGpl) are present in human plasma or serum, but the relative concentration of the ether phospholipids in plasma is very low as compared to those in other tissues. Nowadays, measurement of ether phospholipids in plasma depends on tandem mass spectrometry (LC/MS/MS), but a system for LC/MS/MS is generally too expensive for usual clinical laboratories. Treatment of plasma with phospholipase A_1_ (PLA1) causes complete hydrolysis of diacylphospholipids, but ether phospholipids remain intact. After the treatment of plasma with PLA1, both eEtnGpl and eChoGpl are detected as independent peaks by high-performance liquid chromatography with evaporative light scattering detection (HPLC–ELSD). The same sample used for HPLC–ELSD can be applied to detect eEtnGpl and eChoGpl with electrospray ionization mass spectrometry. Presence of alkylacylphospholipids in both eChoGpl and eEtnGpl in human plasma was indicated by sequential hydrolysis of plasma with PLA1 and hydrochloric acid.

## Introduction

Ether phospholipids constitute a special class of phospholipids characterized by the presence of ether bonds at the* sn*-1 position of the glycerol backbone. There are two types of ether bonds in ether phospholipids: alkyl bonds and alkenyl bonds. Phospholipids with an alkenyl bond (vinyl ether bond) are called plasmalogens [1–5]. Ether phospholipids are found in almost all mammalian tissues, and plasmalogens constitute about 18–20 % of the total phospholipids in cell membranes [1]. Ethanolamine ether phospholipids (eEtnGpl) are much more abundant than choline ether phospholipids (eChoGpl) in animal tissues except heart and skeletal muscle [1, 4]. Plasmalogens are not only structural component of mammalian cell membranes and a reservoir for second messengers but also may be involved in membrane fusion, ion transport, and cholesterol efflux [1–5]. The vinyl ether bond at the* sn*-1 position makes plasmalogens more susceptible to oxidative stress than the corresponding ester-bonded glycerophospholipids. Therefore, plasmalogens may act as antioxidants and protect cells from oxidative stress [1, 4, 5].

Ethanolamine ether phospholipids (eEtnGpl) and choline ether phospholipids (eChoGpl) are found in human serum (plasma), but the relative concentration of the ether phospholipids in the plasma phospholipids are very low as compared to those in other tissues such as leukocytes and erythrocytes. Functions or physiological roles of ether phospholipids in serum (plasma) are not well elucidated, but decreases in plasmalogens in serum (plasma) have been reported in several diseases such as Alzheimer’s disease [6–9], Parkinson’s disease [10], metabolic syndrome [11–13], schizophrenia [14, 15], uremic patients [16], and inflammatory bowel disease [17].

Measurement of the ether phospholipids in human serum (plasma) has been difficult because of its low concentration. Gas chromatography (GC) and/or gas chromatography coupled with mass spectrometry (GC/MS) has been used for measurement of plasmalogens in serum (plasma) by detecting fatty aldehydes (dimethyl acetals) [16, 18], but these methods were an indirect assay for plasmalogens. Nowadays measurement of serum plasmalogens seems to depend on liquid chromatography–tandem mass spectrometry (LC/MS/MS) [6–9, 17, 19–22]. An HPLC method with a flow gamma counter by use of radioactive iodine is reported [8, 23]. However, all of these methods require expensive apparatus and are time-consuming.

Phospholipase A_1_ (PLA1) hydrolyzes ester (acyl) bond at the* sn*-1 position of glycerophospholipids, but it does not act on ether bond at the* sn*-1 position. When serum and/or plasma is treated with PLA1, all of the diacyl phospholipids are completely hydrolyzed; however, ether phospholipids remain intact. The treatment of plasma with PLA1 makes eEtnGpl and eChoGpl detectable as independent peaks in the HPLC with evaporative light scattering detection (HPLC–ELSD). The same sample used for the HPLC method can be applied to liquid chromatography–electrospray ionization mass spectrometry (LC/ESI–MS). HPLC–ELSD and LC/ESI–MS systems are generally less expensive and are easier to maintain than those of LC/MS/MS.

## Materials and Methods

### Materials

Phospholipase A_1_ from *Thermomyces lanuginosus* expressed in *Aspergillus orizae* was purchased from Sigma-Aldrich Co. (Tokyo, Japan). Specific activity of the PLA1 was not recorded. PLA1 from *A. orizae* (10,000–13,000 U/g) was purchased from Mitsubishi Kagaku Foods Co. (Tokyo, Japan). Phosphatidylethanolamine from egg yolk, phosphatidylcholine from bovine liver, and sphingomyelin from bovine heart were purchased from Doosan Serdary Res. Lab. (Toronto, Canada) through Funakoshi Co. (Tokyo, Japan).

### Preparation of Ether Phospholipids in Human Plasma

With informed consent, human venous blood of volunteers who were aged over 70 years (male 13, female 29; age 76 ± 4.6) was drawn into a tube containing heparin, and plasma was separated by using a clinical centrifuge at 1000*g* for 5 min. Plasma was kept at −80 °C until use. PLA1 (Sigma) was diluted with an equal volume of 0.1 M citrate buffer (pH 4.5), and 20 μL of the diluted PLA1 was added to 80 μL of plasma and incubated at 45 °C for 60 min.

### Extraction of Lipids

Lipid extraction after the treatment of plasma with PLA1 was done according to the reported method [24]. Eight hundred microliters of *n*-hexane/isopropanol (3:2, v/v) was added to the PLA1-treated plasma, and after vigorous mixing, it was placed in an ultrasound bath for 5 min. Then 400 μL of Na_2_SO_4_ solution (1 g of anhydrous Na_2_SO_4_ dissolved in 15 mL of water) was added and left for 5 min. Four hundred microliters of the hexane layer was transferred to a new conical Eppendorf tube. Then 400 μL of hexane/isopropanol (7:2, v/v) was added to the lower phase and vigorously mixed, and the hexane layer (300 μL) was recovered. The combined hexane layer was dried under N_2_ gas and stored at −30 °C until use.

In some experiments a chloroform/methanol (1:2, v/v) method was used with some modification from Bligh and Dyer’s method [25]. After treatment of plasma with PLA1 as described, 50 μL of physiological saline solution was added. Then 300 μL of chloroform/methanol (1:2, v/v) was added, vortexed for 1 min, and sonicated for 1 min. After mixing with 100 μL of chloroform, 100 μL of water was added. After brief centrifugation, 160 μL of the lower chloroform phase was transferred to a new tube. Lipids were re-extracted from the remaining lower phase with 100 μL of chloroform, and 100 μL of the lower phase was recovered. The combined chloroform phase was dried under N_2_ gas and stored at −30 °C until use.

### HPLC–ELSD Method

Separation of phospholipid classes including ether phospholipids by HPLC was done by our reported method [26]. The HPLC system was an Agilent 1100 equipped with a four-solvent delivery system, a degasser, an automatic injector, and evaporative light scattering detector. The column was a LiChrosphere 100 Diol (250 × 2 mm), 5 μm (Merck, Germany). Mobile phase A was *n*-hexane/2-propanol/acetic acid (82:17:1, v/v/v) with 0.08 % triethylamine (TEA), and mobile phase B was 2-propanol/water/acetic acid (85:14:1, v/v/v) with 0.08 % TEA. Mobile phase B was 4 % at 0 min, and increased linearly to 37 % in 21 min. The gradient continued from 37 to 85 % in 4 min, then 85 % B was maintained for 1 min. Mobile phase B was decreased to 4 % in 3 min and then 4 % B was maintained for 7 min to precondition the column ready for the next injection. The column temperature was 50 °C and flow rate was 0.4 mL/min. The phospholipid classes were detected by ELSD (Agilent 1900 Infinity) with the following settings: evaporation temperature, 60 °C; sensitivity gain, 6; flow rate of N_2_ gas, 1 L/min. Nebulizer temperature was 30 °C. The lipid extract after PLA1 treatment of plasma was reconstituted with 200 μL of hexane/isopropanol (3:2, v/v), and 20 μL was applied to the HPLC system.

### LC–ESI–MS Method

LC separation of phospholipids classes was done according to the reported method [27]. A Nucleosil diol 100-5-OH column (250 × 3 mm, 5 μm, Machery-Nagel, Germany) was used. Mobile phase A was 53 mM formic acid in acetonitrile (pH 4.5) and mobile phase B was 60 mM ammonium formate and 53 mM formic acid in water (pH 3.64). The flow rate was 0.5 mL/min with gradient elution of 90 % A in 0–2 min, 90–70 % A in 2–15 min, 70 % A in 15–17 min, 70–90 % A in 15–17 min, 90 % A in 5–30 min. The column temperature was 50 °C. Detection of phospholipids was done with an ESI mass spectrometer (Agilent 6120) with selected ion monitoring (SIM) and full scan mode (TIC) in negative ionization mode. The selected ions for eEtnGpl, eChoGpl, and sphingomyelin (cerPCho) are listed in Tables 1 and 2. The SIM ions were selected from ions in peaks of eEtnGpl and eChoGpl of human plasma with full scan mode, and they were picked up from the reports of plasmalogens in human plasma [11–13]. Capillary voltage was 3500 V, scan rate was 1.0/s, nebulizer gas was set at 45 psi, gas flow was 10.0 L/min, source temperature was 350 °C, and full scans were performed from 680 to 950 *m/z*.

**Table 1:** Selected ions in LC/ESI–MS analysis of ether phospholipids in human plasma

	Ion (*m/z*)		Molecular species			Ion (*m/z*)		Molecular species	
eEtnGpl	688.5				eChoGpl	762.5	C16:0/C16:0		
[M − H]^−^	700.5	C16:0/C18:1	C18:0/C16:1	C18:1/C16:0	[M + COOH]^−^	776.5	C16:0/C17:0		
	720.5	C16:0/C20:5				778.8			
	722.5	C16:0/C20:4				786.6	C16:0/C18:2		C18:1/C16:1
	724.5	C16:0/C20:3		C18:1/C18:2		787.5			
	726.5		C18:0/C18:2	C18:1/C18:1		788.6	C16:0/C18:1	C18:0/C16:1	
	728.5		C18:0/C18:1			800.5			C18:1/C17:1
	746.5	C16:0/C22:6		C18:1/C20:5		802.5		C18:0/C17:1	C18:1/C17:0
	748.5	C16:0/C22:5	C18:0/C20:5	C18:1/C20:4		803.5			
	749.5					808.6	C16:0/C20:5		
	750.6	C16:0/C22:4	C18:0/C20:4	C18:1/C20:3		810.6	C16:0/C20:4		
	762.5					812.6	C16:0/C20:3		C18:1/C18:2
	772.6			C18:1/C22:6		813.6			
	773.3					814.6		C18:0/C18:2	C18:1/C18:1
	774.6		C18:0/C22:6	C18:1/C22:5		816.6		C18:0/C18:1	
	775.3					828.5			
	776.7		C18:0/C22:5	C18:1/C22:4		830.6			
	778.8		C18:0/C22:4			834.6	C16:0/C22:6		C18:1/C20:5
	802.5					836.6	C16:0/C22:5	C18:0/C20:5	C18:1/C20:4
	860.6					838.6	C16:0/C22:4	C18:0/C20:4	C18:1/C20:3
						839.5			
						840.7		C18:0/C20:3	
						860.6			C18:1/C22:6
						862.7		C18:0/C22:6	C18:1/C22:5

**Table 2:** Selected ions of PtdEtn, PtdCho, and CerPCho for standard curve for LC/ESI–MS

	Ion (*m/z*)		Molecular species			Ion (*m/z*)	Molecular species	lon (*m/z*)
PtdEtn	714.5	C16:0/C18:2			CerPCho	719.5	C14:0	745.6
[M − H]^−^	716.5	C16:0/C18:1			[M + COOH]^−^	747.5	C16:0	746.5
	738.5	C16:0/C20:4	C18:2/C18:2			749.5	C16:1	748.5
	742.5	C18:0/C18:2	C18:1/C18:1	C16:0/C20:2		775.3	C18:0	776.7
	744.5	C18:0/C18:1				773.3	C18:1	802.5
	764.5	C18:1/C20:4	C16:0/C22:5	C18:0/C20:5		771.3	C18:2	816.6
	766.5	C18:0/C20:4	C16:0/C22:4			803.5	C20:0	830.6
						831.5	C22:0	843.6
						829.6	C22:1	845.6
PtdCho	802.5	C16:0/C18:2				859.3	C24:0	860.6
[M + COOH]^−^	804.5	C16:0/C18:1				857.6	C24:1	935.1
	830.5	C18:0/C18:2	C18:1/C18:1	C16:0/C20:2		855.6	C24:2	
	832.5	C18:0/C18:1				858.3		
	854.5	C18:1/C20:4	C16:0/C22:5	C18:0/C20:5		720.5		
	856.5	C18:0/C20:4	C16:0/C22:4			733.5		

The same sample used for the HPLC–ELSD method was applied to the LC/ESI–MS method.

### Acid Hydrolysis of Ether Phospholipids

Acid hydrolysis of ether phospholipids with hydrochloric acid (HCl) was done after PLA1 treatment of total lipids [28, 29]. Briefly, the dried lipid extract after the treatment with PLA1 was dissolved in 300 μL of methanol/chloroform/water (2:1:1, v/v/v), then 150 μL of 1 N HCl and 150 μL of methanol were added. After vigorous mixing, the solution was incubated at room temperature for 1 h. Then, 150 μL each of chloroform and water were added to the solution. The solution was centrifuged at 1000*g* for 5 min. The chloroform layer was dried under N_2_ gas (HCl solution method). The acid-hydrolyzed lipids were resuspended in hexane/isopropanol (3:2, v/v) and subjected to the HPLC–ELSD and LC/ESI–MS methods.

Another acid hydrolysis method for the ether phospholipids was also used [30]. The dried lipids after the treatment with PLA1 in the test tubes were inverted over five drops of 12 N HCl for 5 min (HCl fume method) [30]. The lipids were extracted with hexane/isopropanol (3:2, v/v) as described earlier (“Extraction of Lipids”). Lipids were reconstituted in hexane/isopropanol (3:2, v/v) and subjected to the HPLC–ELSD method.

## Results and Discussion

### Hydrolysis of Plasma with PLA1

The brochure from the provider reported that the optimum temperature and optimum pH for PLA1 activity are 45 °C and pH 4.5, respectively. Therefore, we diluted PLA1 with 0.1 M citrate buffer (pH 4.5) and the incubation of plasma with PLA1 was done at 45 °C.

**Fig. 1:**
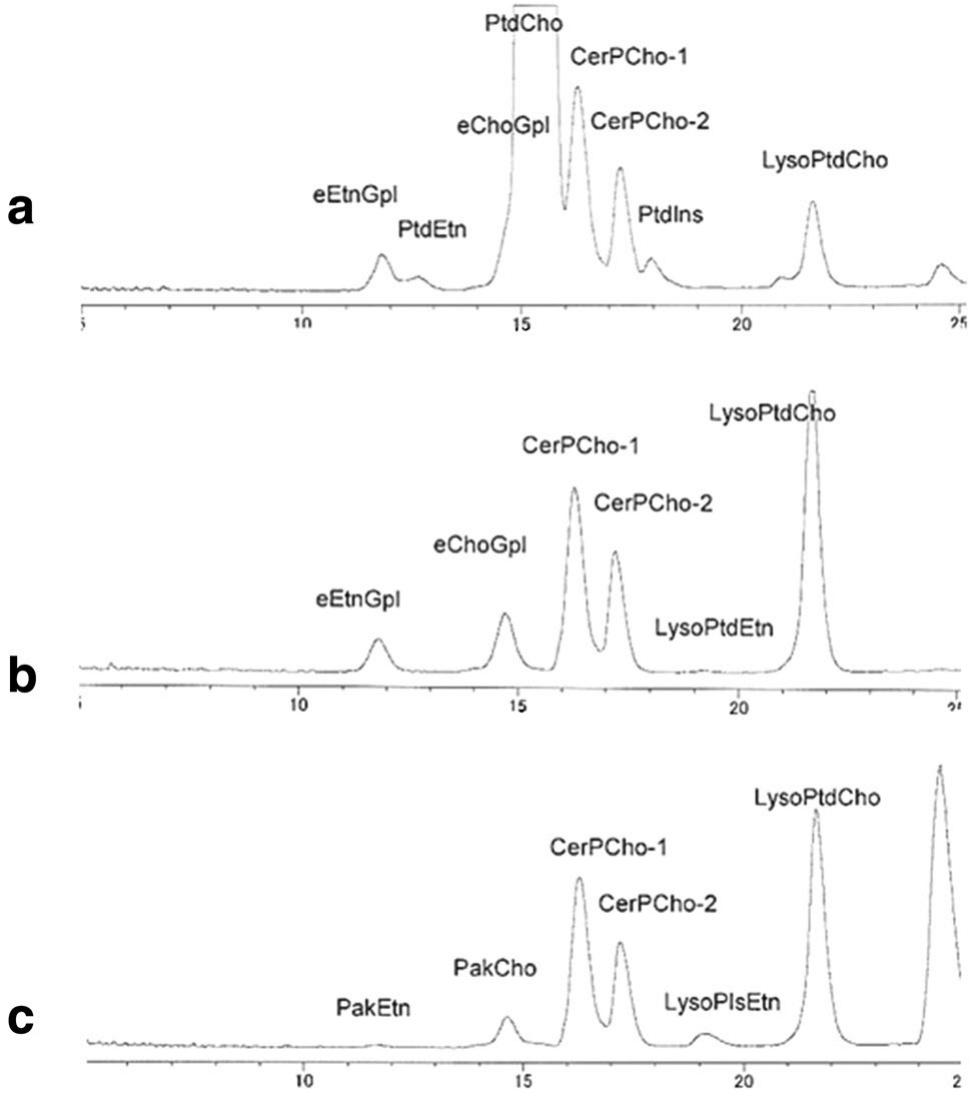
HPLC–ELSD chromatograms of **a** total phospholipids of human plasma, **b** after phospholipase A_1_ (PLA1) treatment of plasma, **c** after acid hydrolysis of PLA1-treated plasma. LysoPlsEtn, which was a product of acid hydrolysis of PlsEtn, was observed. Lipids were extracted with chloroform/methanol (1:2, v/v). *PtdEtn* phosphatidylethanolamine, *PtdCho* phosphatidylcholine, *PtdIns* phosphatidylinositol, *LysoChoGpl* lysophosphatidylcholine, *eEtnGpl* ethanolamine ether phospholipid, *eChoGpl* choline ether phospholipids, *PakCho* choline alkylacylphospholipid, *PakEtn* ethanolamine alkylacylphospholipid

Figure 1 shows HPLC–ELSD chromatograms (a) before and (b) after the treatment of plasma with PLA1 at 45 °C for 60 min. Diacylglycerophospholipids disappeared completely on treatment with PLA1.

PLA1 purchased from Mitsubishi Kagaku Food Co. was suspended in 0.1 M citrate buffer (50 mg/mL). Forty microliters of the enzyme solution was added to 80 μL of plasma and incubated at 45 °C for 60 min.

**Fig. 2:**
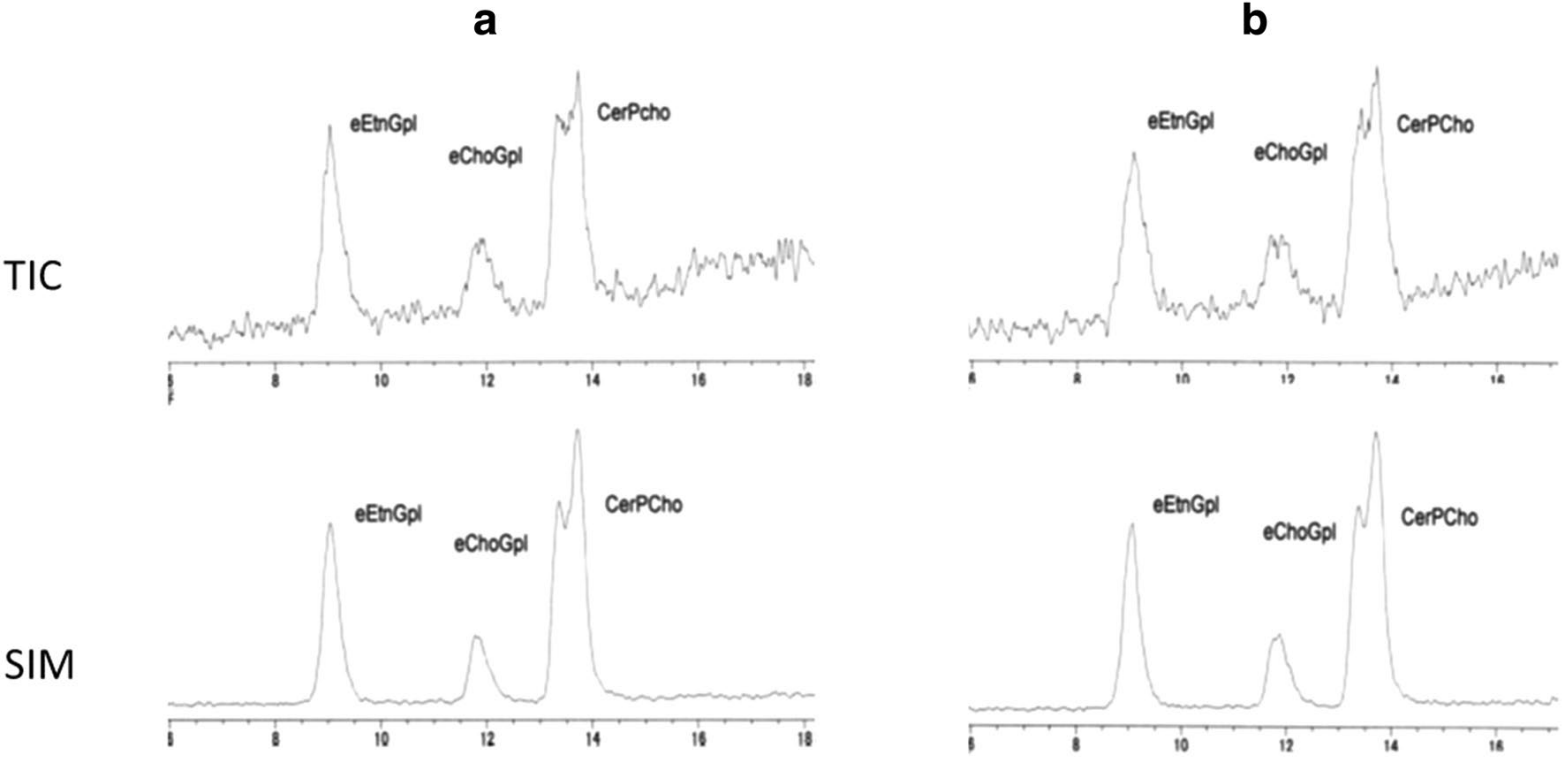
LC–ESI–MS chromatograms of plasma treated with PLA1 from **a** Sigma Co, **b** Mitubishi Kagaku Food Co. The same human plasma was used for hydrolysis with PLA1 from different sources. All of the peak areas and shapes were identical. Total ion current mode (TIC) and selected ion mode (SIM) are shown, but the baseline of SIM was clearer than that of TIC. For abbreviations, see Fig. 1

The two different PLA1 which were obtained from different sources were applied to the same human plasma, and the results showed identical chromatograms of ESI detection (Fig. 2a, b).

**Fig. 3:**
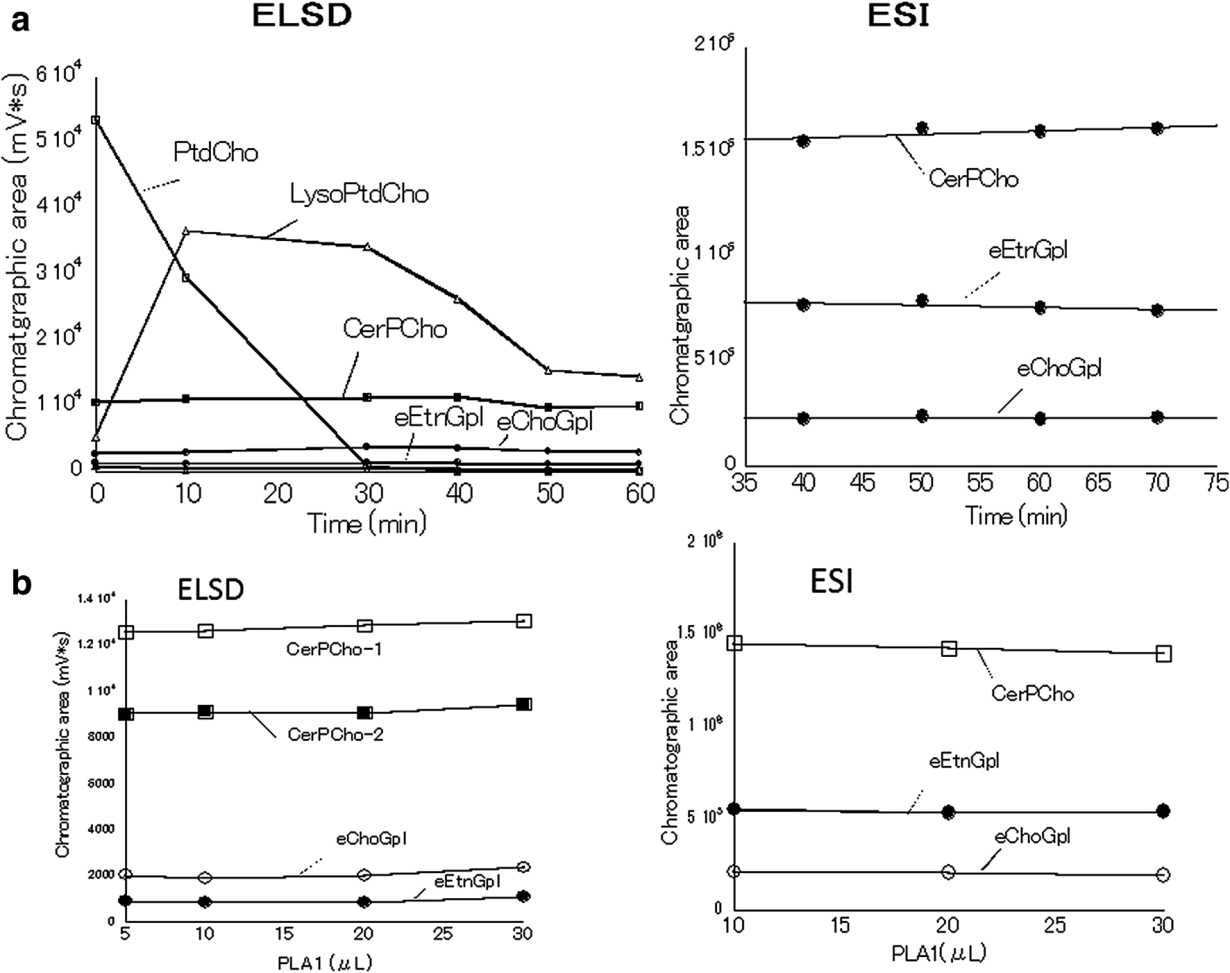
**a** Time courses of main phospholipids in human plasma during 60 min incubation with PLA1. PtdCho was completely hydrolyzed within 40 min. LysoPtdCho decreased gradually until 50 min after rapid increase within 10 min. However, ePtdEtn, ePtdCho, and CerPCho remained unchanged for 60 min. Lipids were extracted with chloroform/methanol (2:1, v/v). **b** Levels of the ether phospholipids and CerPCho were constant with different amounts of PLA1 during 60 min incubation at 45 °C. Values are the mean of duplicated determinations. See Fig. 1 for other abbreviations

The time courses of the changes in each main phospholipid in plasma during incubation of plasma with PLA1 at 45 °C for 60 min are shown in Fig. 3. Phosphatidylcholine (PtdCho), which is the most abundant phospholipid in plasma, was hydrolyzed completely within 40 min (Fig. 3a). Lysophosphatidylcholine (LysoPtdCho), which was the product from hydrolysis of PtdCho by PLA1, increased rapidly within 10 min and then decreased gradually until 50 min (Fig. 3a). On the other hand, ether phospholipids (eEtnGpl and eChoGpl) and sphingomyelin (CerPCho) remained unchanged for 60 min incubation at 45 °C (Fig. 3a). Furthermore, the levels of the ether phospholipids (eEtnGpl and eChoGpl) and sphingomyelin (CerPCho) were constant by incubation with different amounts of PLA1 for 60 min at 45 °C (Fig. 3b).

### HPLC (LC) Method

The HPLC–ELSD method can separate eEtnGpl and eChoGpl as well as all other phospholipids classes usually found in mammalian tissues by a single chromatographic run [26]. However, in the case of human plasma, the HPLC can separate ethanolamine ether phospholipids (eEtnGpl) from phosphatidylethanolamine (PtdEtn), but separation of choline ether phospholipid (eChoGpl) from phosphatidylcholine (PtdCho) was incomplete, because human plasma contains exceptionally high amounts of phosphatidylcholine as compared to the other tissues (Fig. 1a).

Treatment of plasma with PLA1 afforded eChoGpl as an independent peak and made quantification of eChoGpl with the HPLC method more accurate (Fig. 1b). The PLA1 treatment of plasma did not hydrolyze sphingomyelin as well as the ether phospholipids (Fig. 1b); therefore, we also quantified sphingomyelin (CerPCho) in parallel with eEtnGpl and eChoGpl. Authentic sphingomyelin as well as plasma sphingomyelin showed two large peaks by the HPLC–ELSD method (Fig. 1).

Any HPLC methods which can separate EtnGpl, CholGpl, and CerPCho can be used for the present method, because the treatment of plasma with PLA1 leaves only ether phospholipids and sphingomyelin. The HPLC method used for LC/ESI–MS was different from that of the HPLC–ELSD method, because we were advised by the manufacturer of the ESI system that use of trimethylamine (TEA) in the mobile phase of ESI detection should be avoided as much as possible. Detection of ether phospholipids with ESI increases sensitivity as compared to that with ELSD (Figs. 1, 2). We monitored both selected ion monitoring (SIM) and full scan mode (TIC) in negative ionization mode in the LC/ESI–MS method. The peak shapes were almost identical in TIC and SIM, but the baselines of chromatograms from the SIM mode were clearer than those from the TIC mode (Fig. 2). Therefore, data from the SIM mode were used for calculation of phospholipids (Table 2).

**Fig. 4:**
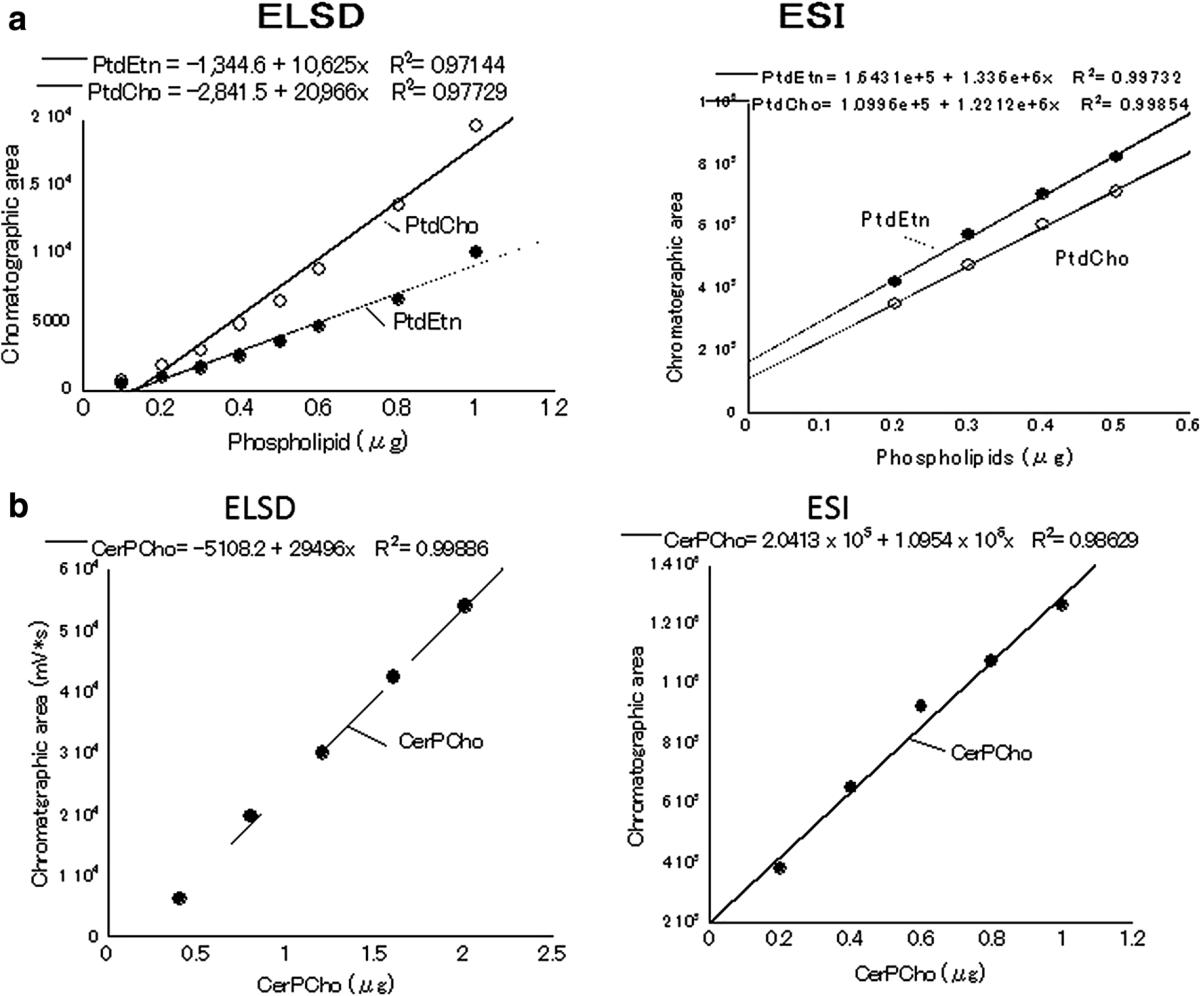
Standard curves for diacylphospholipids (PtdEtn, PtdCho) and sphingomyelin (CerPCho) for ELSD detection and ESI detection. All linear regressions (*R* ^2^) were above 0.97. See Fig. 1 for abbreviations

Because standard curves for phospholipids by detection with ELSD do not show linearity [31–33], we used lineal regression curves (*R*^2^ > 0.97) for calculation, and eEtnGpl and eChoGpl were calculated as PtdEtn and PtdCho, respectively (Fig. 4). For calculation in micromolar units, the following molecular weight (MW) was used: PtdCho = 790.2, PtdEtn = 748.1, CerPCho = 703. The amounts of ether phospholipids in human plasma determined by the present methods are shown in Table 3. The ranges of values of eEtnGpl and eChoGpl were in accordance with the reported values [8, 22, 23]. Data of eEtnGpl and eChoGpl from ELSD detection were almost identical to those from ESI detection. However, the mean value of CerPCho from ELSD was lower than that of LC/MS (Table 3). These discrepancies may be caused by the difference of detection method (ELSD and ESI). We learned from our experiences that peak areas by ELSD detection change considerably with differences of gain, nebulizer temperature, or evaporation temperature.

**Table 3:** Amounts of ether phospholipids and sphingomyelin in human plasma (μM)

	LC/ESI–MS	HPLC–ELSD
eEtnGpl	65.1 ± 29.8	62.1 ± 19.1
eChoGpl	65.9 ± 15.1	75.3 ± 22.4
CerPCho	326.3 ± 74.4	228.2 ± 48.3

Values are mean ± standard deviation (*n* = 42)

### Lipid Extraction

**Fig. 5:**
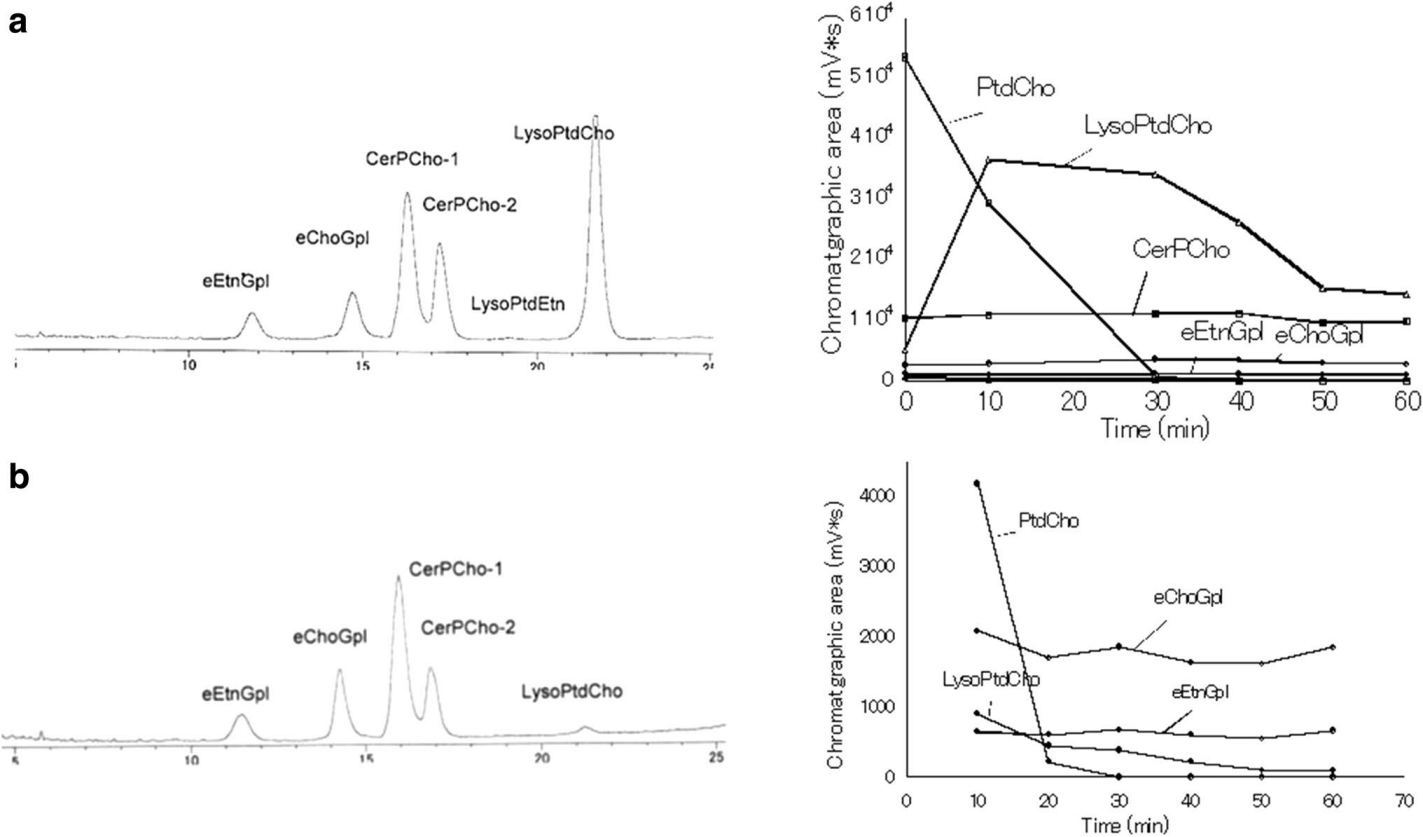
Apparent differences of lysophospholipids by the different lipid extraction methods. **a** Chloroform/methanol (1:2, v/v) method. **b** Hexane/isopropanol (3:2, v/v) method. Samples (plasma) used were different from each other. For abbreviations, see Fig. 1

The recoveries of lysophospholipids were different according to the lipid extraction method. The chloroform/methanol method retained large amounts of lysophospholipids (Figs. 1, 5a), but the hexane/isopropanol method (3:2, v/v) removed almost all of the lysophospholipids (Fig. 5b). However, the amounts of the ether phospholipids and sphingomyelin were not different between the two methods (Fig. 5).

### Presence of Alkylacylphospholipids in Human Plasma

**Fig. 6:**
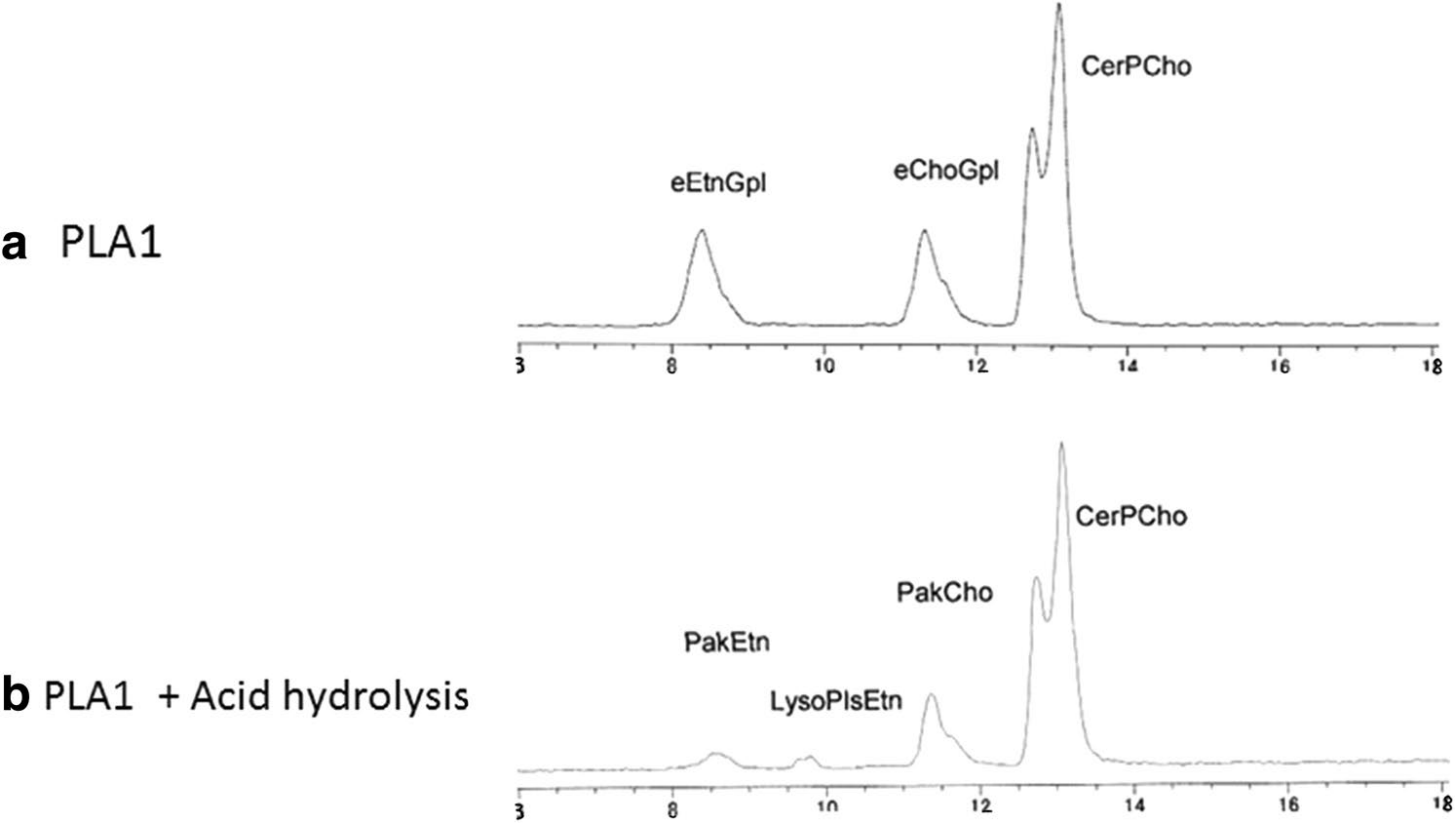
LC chromatograms with ESI detection. **a** Chromatogram of plasma after hydrolysis with PLA1. **b** Chromatogram after acid hydrolysis of PLA1-treated plasma. Peak of lyso-ethanolamine plasmalogen (LysoPlsEtn) which was a product of acid hydrolysis of PlsEtn was observed. For abbreviations, see Fig. 1

To our knowledge, few reports have mentioned the presence of alkylacylphospholipids in human plasma or serum. After the hydrolysis of plasma with PLA1, samples were further hydrolyzed with HCl (HCl solution method). We found that some of the peaks of ether phospholipids remained after the hydrolysis with HCl in all of the six samples examined (Fig. 1c), indicating that part of the ether phospholipids in human plasma is alkylacylphospholipids (PakEtn and PakCho). Furthermore, LysoPlsEtn, which was a product of acid hydrolysis of PlsEtn, was observed in the chromatograms (Figs. 1c, 6b) Because sphingomyelin was not changed by the acid hydrolysis, each area of the ether phospholipids (eChoGpl and eEtnGpl) and each area of PakEtn and PakCho were calculated on the basis of sphingomyelin (Table 3). PakCho was indicated to represent more than 60 % (67.5 ± 3.5 %, *n* = 6) of choline ether phospholipids (eChoGpl) by ESI detection, and it was indicated to represent more than 50 % (55.9 ± 2.7 %, *n* = 6) by ELSD detection. We also detected PakEtn: (19.8 ± 3.3 %, *n* = 6) by ESI and (15.3 ± 5.0 %, *n* = 6) by ELSD. The presence of alkylacylphospholipids in human plasma was also confirmed by another acid hydrolysis method of ether phospholipid by using fumes of 12 N HCl for 5 min (HCl fume method) [30]. Amounts of PakCho detected after the HCl fume method were almost the same as those after the HCl solution method by detection with ELSD (Table 4); however, amounts of PakEtn detected after the HCl fume method were much lower than that after the HCl solution method (Table 4). Furthermore, no PakEtn was detected in three samples out of six samples after the HCl fume method.

**Table 4:** Alkylacylphospholipids in human plasma detected by different methods (% of each ether phospholipid)

	HCI solution method		HCI fume method
	LC–ESI–MS	HPLC–ELSD	HPLC–ELSD
PakEtn	19.8 ± 5.2	15.3 ± 5.0	8.8 ± 9.9
PakCho	67.5 ± 3.3	55.9 ± 2.8	59.2 ± 12.9

Values are mean ± standard deviation (*n* = 6)

## Conclusion

We developed an HPLC–ELSD method for measurement of ether phospholipids in human plasma after hydrolysis of plasma with PLA1. The same sample can be used for both HPLC–ELSD and LC/ESI–MS. The HPLC–ELSD and LC/ESI–MS systems are generally less expensive and much easier to maintain than LC/MS/MS. Any HPLC method which can separate PtdEtn, PtdCho, and CerPCho can be used for the present methodology, because treatment of sample with PLA1 leaves only ether phospholipids and sphingomyelin. Furthermore, the presence of alkylacylphospholipids (PakEtn and PakCho) in human plasma was observed by sequential hydrolysis of plasma with PLA1 and HCl. These methodologies including the method for differentiation of phospholipid subclasses may be applied to tissues other than human plasma.

## Abbreviations

CerPCho: Sphingomyelin
ELSD: Evaporative light scattering detection
ESI: Electrospray ionization
eChoGpl: Choline ether phospholipid
eEtnGpl: Ethanolamine ether phospholipid
HPLC: High-performance liquid chromatography
LC: Liquid chromatography
LysoPlsEtn: Lyso-ethanolamine plasmalogen
LysoPtdCho: Lysophosphatidylcholine
MS: Mass spectrometry
PakCho: 1-*O*-Alkyl-2-acyl-*sn*-glycero-3-phosphocholine
PakEtn: 1-*O*-Alkyl-2-acyl-*sn*-glycero-3-phosphoethanolamine
PLA1: Phospholipase A_1_
PlsCho: Choline plasmalogen
PlsEtn: Ethanolamine plasmalogen
PtdEtn: Phosphatidylethanolamine
PtdCho: Phosphatidylcholine
